# The association of HLA-DQB1, -DQA1 and -DPB1 alleles with anti- glomerular basement membrane (GBM) disease in Chinese patients

**DOI:** 10.1186/1471-2369-12-21

**Published:** 2011-05-13

**Authors:** Huan Luo, Min Chen, Zhao Cui, Rui Yang, Peng-Cheng Xu, Xu-Jie Zhou, Ming-Hui Zhao

**Affiliations:** 1 Renal Division, Department of Medicine, Peking University First Hospital; Institute of Nephrology, Peking University; Key Laboratory of Renal Disease, Ministry of Health of China; Beijing 100034, China

**Keywords:** Anti-GBM disease, HLA-DPB1*0401, Chinese

## Abstract

**Background:**

Human leukocyte antigen (HLA) alleles are associated with many autoimmune diseases, including anti-glomerular basement membrane (GBM) disease. In our previous study, it was demonstrated that HLA-DRB1*1501 was strongly associated with anti-GBM disease in Chinese. However, the association of anti-GBM disease and other HLA class II genes, including HLA-DQB1, -DQA1,-DPB1 alleles, has rarely been investigated in Asian, especially Chinese patients. The present study further analyzed the association between anti-GBM disease and HLA-DQB1, -DQA1, and -DPB1 genes. Apart from this, we tried to locate the potential risk amino acid residues of anti-GBM disease.

**Methods:**

This study included 44 Chinese patients with anti-GBM disease and 200 healthy controls. The clinical and pathological data of the patients were collected and analyzed. Typing of HLA-DQB1, -DQA1 and -DPB1 alleles were performed by bi-directional sequencing of exon 2 using the SeCoreTM Sequencing Kits.

**Results:**

Compared with normal controls, the prevalence of HLA-DPB1*0401 was significantly lower in patients with anti-GBM disease (3/88 vs. 74/400, p = 4.4 × 10^-4^, pc = 0.039). Comparing with normal controls, the combination of presence of DRB1*1501 and absence of DPB1*0401 was significantly prominent among anti-GBM patients (p = 2.0 × 10^-12^, pc = 1.7 × 10^-10^).

**Conclusions:**

HLA-DPB1*0401 might be a protective allele to anti-GBM disease in Chinese patients. The combined presence of DRB1*1501 and absence of DPB1*0401 might have an even higher risk to anti-GBM disease than HLA-DRB1*1501 alone.

## Background

Anti-glomerular basement membrane (GBM) disease, defined by the presence of autoantibodies in the circulation against α3 chain non-collagen 1 domain of type IV collagen [α3(IV)NC1] [1], is a severe autoimmune disease. It manifests as rapidly progressive glomerulonephritis; when accompanied by alveolar hemorrhage, it is termed Goodpasture's disease.

Human leukocyte antigen (HLA) alleles, located on the short arm of chromosome 6, have been well known to be associated with most autoimmune diseases [2]. HLA genes encode numerous molecules including the HLA class I and II molecules, which have immunological functions. It was reported that anti-GBM disease was positively associated with HLA-DRB1*1501 and negatively associated with HLA-DRB1*07 in Caucasian population [3]. In Asian populations, HLA-DRB1*1501 was also considered as a risk allele for Japanese [4] and Chinese patients [5]. These data suggested that HLA-DRB1*1501 is a common risk allele for anti-GBM disease in various populations. However, the association of anti-GBM disease and HLA class II genes, including HLA-DRB1, -DQB1, -DQA1, and -DPB1 alleles, has rarely been investigated in Asian, especially Chinese patients [4,5]. Since our previous study has located the risk allele of HLA-DRB1 [5], to better understand the genetic background of this disease and prepare for the study at the level of peptide, in the current study, we further investigated the distribution and clinical association of HLA-DQB1, -DQA1 and -DPB1. Moreover, we tried to locate the potential risk amino acid residues of anti-GBM disease.

## Methods

### Patients

Forty-four patients with anti-GBM disease, who were diagnosed at Renal Division, Peking University First Hospital, from 1996 to 2007, were included in this study. Anti-GBM disease was defined as the patient had glomerulonephritis and/or pulmonary hemorrhage, and patient's serum contained circulating anti-GBM antibodies [6]. The onset of the disease was judged by renal or extra-renal signs and symptoms of anti-GBM disease, or abnormalities related to anti-GBM disease were detected by various examinations, including hemoptysis, oliguria or anuria, hematuria or elevated serum creatinine [6,7]. All the 44 patients received renal biopsy. Clinical and pathological data were collected at the time of renal biopsy. Two hundred ethnically matched healthy blood donors were employed as normal controls. The research was in compliance of the declaration of Helsinki and approved by the ethic committee of the local hospital. Informed consent was obtained from each patient.

### Detection of serum anti-GBM autoantibodies

Anti-GBM autoantibodies were measured by ELISA using bovine α(IV)NC1 as the solid phase antigen, which was described previously [8]. The results were expressed as relative absorbance value to a percentage of a known positive control serum, and values greater than 13% were regarded as positive.

### Samples

Peripheral blood samples (10 ml) from patients with anti-GBM disease and normal controls were collected in EDTA. Genomic DNA was obtained from peripheral blood leukocytes with a salting-out procedure [9].

### Sequence based typing

Typing of HLA-DQB1, -DQA1 and -DPB1 alleles were performed by bi-directional sequencing of exon 2 using the SeCoreTM Sequencing Kits (Invitrogen, Brown Deer, WI, USA).

### Statistical analysis

The difference in the frequencies of HLA alleles in disease samples and controls was compared using the Chi-square test or Fisher's exact test as appropriate. To compare the HLA alleles of subjects stratified by various demographic and clinical parameters, Chi-square test, Fisher's exact test, or nonparametric test was used as appropriate. Bonferroni correction was applied to correct p-value (p corrected, pc). It was considered significant difference if the pc value was less than 0.05. The statistical analysis was performed in SPSS statistical software package (version 11.0, Chicago, Ill, USA). The evaluation at the amino acid level including the examination of polymorphic amino acid residue, pocket of amino acid, zygosity and tests for association, interaction, and linkage disequilibrium among amino acid epitopes of the same HLA molecule or between HLA isotypes were conducted by SKDM software program [10].

## Results

### Demographic and clinicopathological features

Among the 44 patients with anti-GBM disease, 30 were male and 14 were female. The median age of the 44 patients was 27 (range 13-82) years old on diagnosis. Sixteen out of 44 patients had pulmonary hemorrhage. All of the patients had hematuria and proteinuria. 17/44 (38.6%) patients had anuria or oliguria. On diagnosis, the level of serum creatinine was 765.4 ± 388.7 μmol/L. Renal biopsy was performed in all the 44 patients. 41/44 (93.8%) patients had crescent formation in more than 50% of the glomeruli and 30 (68.2%) had crescent formation in more than 85% of the glomeruli in the renal specimen. Direct immunofluorescence examination was performed in 35 cases. All of them showed linear or fine granular IgG and/or C3 deposition along glomerular capillary wall. Outcome data were available for 40 out of the 44 patients. At the end of one year after diagnosis, only 7/40 (17.5%) patients were dialysis-independent, and 33/40 (82.5%) patients were dialysis-dependent or died.

### HLA-DQB1, -DQA1 and -DPB1 alleles and their association with anti-GBM disease

The frequencies of each HLA-DQB1, -DQA1 and -DPB1 allele for the 44 patients with anti-GBM disease and 200 ethnically matched healthy controls were determined by sequence based typing. A total of 9 HLA-DQB1 alleles, 9 HLA-DQA1 and 34 HLA-DPB1 alleles typed in our study. Compared with normal controls, the prevalence of HLA-DPB1*0401 was significantly lower in patients with anti-GBM disease (3/88 vs. 74/400, p = 4.4 × 10^-4^, pc = 0.039) (Table 1). However, the age (transformed by log10) of HLA-DPB1*0401 positive patients was significantly younger than that of DPB1*0401 negative patients (p = 0.011). Besides, the proportion of patients having hemoptysis was significantly higher in patients with DPB1*0401 than that in patients without DPB1*0401 (p = 0.042) (Table 2). There was no significant difference between these patients and normal controls on other HLA alleles. No significant difference of gender, age, level of anti-GBM autoantibodies, serum creatinine, or other clinical and pathological parameters was found between anti-GBM patients with and without HLA-DPB1*0401 (Table 2).

**Table 1:** Distribution of HLA-DQB1, -DQA1 and DPB1 alleles in patients with anti-GBM disease and normal controls

HLA alleles	Anti-GBM disease n × 2 = 88	Normal controls n × 2 = 400	p	pc	OR	95% CI	HLA alleles	Anti-GBM disease n × 2 = 88	Normal controls n × 2 = 400	p	pc	OR	95% CI
DQB1*0201	17	95	0.37		0.77	0.43-1.37	DPB1*0801	3	1	0.02	1.76	14.08	1.45-137.03
DQB1*0301	15	45	0.13		1.62	0.86-3.06	DPB1*0901	4	3	0.022	1.94	6.3	1.39-28.68
DQB1*0302	2	13	1		0.69	0.15-3.12	DPB1*1101	0	1	1			
DQB1*0303	7	39	0.6		0.8	0.35-1.85	DPB1*1301	4	5	0.06		3.76	0.99-14.31
DQB1*0401	2	8	0.7		1.14	0.24-5.46	DPB1*1302	0	1	1			
DQB1*0501	13	45	0.36		1.37	0.70-2.66	DPB1*1601	1	4	1		1.14	0.13-10.31
DQB1*0502	3	24	0.44		0.55	0.16-1.88	DPB1*1602	0	1	1			
DQB1*0503	8	37	0.96		0.98	0.44-2.19	DPB1*1701	0	3	1			
DQB1*0602	21	94	0.94		1.02	0.59-1.75	DPB1*1801	1	7	1		0.65	0.078-5.31
DQA1*0101	1	17	0.22		0.26	0.034-1.97	DPB1*2001	2	4	0.3		2.3	0.42-12.77
DQA1*0102	19	148	0.006	0.53	0.47	0.27-0.81	DPB1*2002	0	2	1			
DQA1*0103	2	12	1		0.75	0.17-3.42	DPB1*2101	0	1	1			
DQA1*0201	16	36	0.011	0.97	2.25	1.18-4.27	DPB1*2301	0	3	1			
DQA1*0301	26	92	0.19		1.4	0.84-2.35	DPB1*2401	0	3	1			
DQA1*0302	3	3	0.075		4.67	0.93-23.54	DPB1*2402	3	5	0.16		2.79	0.65-11.89
DQA1*0401	1	12	0.48		0.37	0.048-2.90	DPB1*2601	3	4	0.11		3.49	0.77-15.90
DQA1*0501	19	70	0.37		1.3	0.73-2.29	DPB1*3301	13	44	0.32		1.4	0.72-2.73
DQA1*0601	1	10	0.7		0.45	0.057-3.55	DPB1*3501	0	3	1			
DPB1*0101	1	6	1		0.76	0.090-6.35	DPB1*4001	1	0	0.18			
DPB1*0201	24	98	0.59		1.16	0.69-1.95	DPB1*4501	0	3	1			
DPB1*0301	8	29	0.56		1.28	0.56-2.90	DPB1*4801	0	1	1			
DPB1*0302	1	3	0.55		1.52	1.16-14.80	DPB1*5201	0	1	1			
DPB1*0401	3	74	4.4 × 10^-4^	0.039	0.16	0.048-0.51	DPB1*5401	0	1	1			
DPB1*0402	15	82	0.46		0.8	0.44-1.46	DPB1*5701	0	2	1			
DPB1*0601	1	2	0.45		2.29	2.21-25.51	DPB1*6601	0	1	1			
DPB1*0602	0	1	1				DPB1*8601	0	1	1			

[Abbreviations] pc, P corrected; OR, odds ratio; CI, confident interval.

**Table 2:** Clinical and pathological characteristics of patients with anti-GBM disease, categorized by the presence of HLA gene

	DPB1*0401		
	positive	negative	p value
Gender (male/female)	1/2	29/12	0.23
Age (transformed by log10), mean ± standard deviation	1.37 ± 0.05	1.50 ± 0.19	0.011
Level of anti-GBM antibodies (%), mean ± standard deviation	71.53 ± 61.58	81.43 ± 46.63	0.73
Hemoptysis	3/3	13/41	0.042
Oliguria or anuria	1/3	16/41	1.00
Interval between onset and diagnosis (days), mean ± standard deviation	68.33 ± 31.34	62.87 ± 52.83	0.86
Scr (μmol/L), mean ± standard deviation	1024.33 ± 362.41	746.49 ± 387.91	0.24
Percentage of crescents in glomeruli, median (1st and 3rd quartile)	100 (87.5, 100)	94.87 (67.69, 100)	0.45
Dialysis-dependent or died	3/3	31/37	1.00

[Abbreviations] Scr: serum creatinine.

### The combined analysis of HLA-DRB1 and -DPB1 alleles

When we analyzed our HLA-DPB1 typing data together with HLA-DRB1 typing data from our previous study [5], we found that 2 patients and 19 controls had both HLA-DRB1*1501 and DPB1*0401 present. For those characterized by the combined presence of DRB1*1501 and absence of DPB1*0401, 32 patients and 39 controls were identified. Comparing with controls, the prevalence of this combination was extremely prominent among anti-GBM patients (p = 2.0 × 10^-12^, pc = 1.7 × 10^-10^, OR = 11.01, 95% CI 5.2-23.31).

### The evaluation at the amino acid level

Significant residue was found neither for HLA-DRB1 nor in HLA-DRB1 pockets. For HLA-DPB1, its productions phenylalanine at position 35 (DPB1_F-35) and lysine at position 69 (DPB1_K-69) were observed in decreased frequencies among anti-GBM disease, but their difference was not significant after correction (Table 3). Besides, the evaluations of pocket residues, zygosity analysis and interaction analysis about DPB1_F-35 and K-69 found no significant difference between patients and controls.

**Table 3:** Evaluation at the amino acid level by SKDM

HLA-DPB1 Residues						
Alls	Pos	AA	Assoc	p	pc	OR
0401	35	F	-	0.031	1	0.04
0401	69	K	-	0.0067	0.31	0.28

HLA-DPB1 Pocket Residues				pc		

Pocket 4 (Pos:13,69,76,68,72,24)				0.17		
Pocket 7 (Pos: 26,59,69,45,65)				0.17		
Pocket 9 (Pos: 9,58,55,35,36)				0.83		

[Abbreviations] Alls, alleles; Pos, position; AA, amino acid; Assoc, association; pc, P corrected; OR, odds ratio; F, phenylalanine; K, lysine.

## Discussion

The current study analyzed the distribution of HLA class II alleles in patients with anti-GBM disease and their potential significance. For HLA class II loci, HLA-DRB1 and -DPB1 encode relatively more variable gene products for HLA-DR and -DP molecules respectively, while both HLA-DQB1 and -DQA1 are variable in human population. Besides, previous studies have located some HLA-DRB1, -DQB1 and -DPB1 alleles with association with anti-GBM disease in Caucasian as well as Asian population [5,11,12]. But in Chinese patients, few studies have been done in this topic [5]. Therefore, we choose to type HLA-DQB1, -DQA1 and -DPB1 loci in this study, on the basis of our previous study on HLA-DRB1 [5].

Our typing results indicated that HLA-DPB1*0401 might be non-predisposing on anti-GBM disease. We stratified by the presence of DPB1*0401 on patients with anti-GBM disease and tried to investigate how this allele has its protective influence on clinical and pathological characteristics of patients. However, we found that patients with HLA-DPB1*0401 were younger and were more likely to have hemoptysis. Since there were only three patients with positive HLA-DRB1*0401, larger sample size is needed to investigate the association between this allele and the disease.

Our previous study [5] has located HLA-DRB1*1501 as a risky allele to anti-GBM disease in the same population. When we analyzed the combined presence of DRB1*1501 and absence of DPB1*0401, we found this combination had an even higher risk to anti-GBM disease (p = 2.0 × 10^-12^) than HLA-DRB1*1501 alone (p = 1.6 × 10^-7^). To further investigate how these alleles have their influence on disease, we used SKDM software to evaluate their productions at amino acid level. However, no amino acid with significant difference was found by this evaluation. Since little is known about the relation between HLA-DP and HLA-DR, it is difficult to know how these two alleles interact with each other on molecular level in the pathogenesis of anti-GBM disease.

Previous studies have focused on association between HLA-DR and -DQ genes and their haplotype with anti-GBM disease [3,11,12]. The single allele DQB1*0302, haplotypes DQB1*0602-DRB1*1501 and DQB1*0201-DRB1*0301 were identified as risk alleles [3,11,12], while HLA-DQB1*0501 was considered as a protective allele [3,12]. However, these potential associations were not observed in our study. Actually, no HLA-DQ allele was found to be significantly associated with patients with anti-GBM disease in our study.

HLA class II alleles have been demonstrated a connection with many autoimmune diseases [13-15]. Nevertheless the mechanism underneath is still unknown. HLA association in anti-GBM disease is believed to reflect the ability of certain class II molecules to bind and present peptides derived from the autoantigen to T helper cells[12]. Although at amino acid level, our study showed no significance, theories from other studies may offer us some clues. As far as we have known, the strong positive association with DRB1*1501 [4,5,11,12] as well as negative associations with DRB1*01 and DRB1*07 [3] were found [12,16,17] in many studies. According to above findings, Phelps et al. [17] suggested that DR1/7 (encoded by DRB1*01/07) could protect by capturing α3(IV)NC1 peptides and preventing their display bound to DR15. Judging from this, we speculate that the similar protective mechanism might happen to HLA-DPB1*0401. We suppose that the beta chain of HLA-DP produced by DPB1*0401 prevents peptide such as α3(IV)NC1 from binding DR15, which leads to the disease. Nonetheless, the exact mechanism requires further research to confirm.

## Conclusions

In conclusion, HLA-DPB1*0401 might be a protective allele to anti-GBM disease in Chinese patients. The combined presence of DRB1*1501 and absence of DPB1*0401 might have an even higher risk to anti-GBM disease than HLA-DRB1*1501 alone.

## Competing interests

None declared.

## Authors' contributions

HL collected samples, carried out the study, analyzed the data and drafted the manuscript. MC designed and directed the study, drafted and revised the manuscript. CZ collected samples and clinical and pathological data. RY helped to draft the manuscript. PCX and XJZ helped to draft the manuscript and perform the statistical analysis. MHZ participated the designing and direction. All authors read and approved the final manuscript.

## Pre-publication history

The pre-publication history for this paper can be accessed here:

http://www.biomedcentral.com/1471-2369/12/21/prepub
